# A Novel Sub-Bottom Profiler and Signal Processor

**DOI:** 10.3390/s19225052

**Published:** 2019-11-19

**Authors:** Cheng Tan, Xuebo Zhang, Peixuan Yang, Miao Sun

**Affiliations:** 1 Aerospace Technology Institute of CARDC, Aerodynamics Research and Development Center, Mianyang 621000, China; 2 Laboratory of Underwater Acoustics, Zhanjiang 524000, China; 3 Pan-wisdom Education, Lanzhou 730050, China; 4 China Railway Twenty-one Bureau Group Corporation, Lanzhou 730000, China

**Keywords:** Mills cross configuration, synthetic aperture technique, motion compensation, imaging algorithm

## Abstract

In this paper, we introduce a novel sub-bottom profiler, making good use of the Mills cross configuration of multibeam sonar and synthetic aperture techniques of the synthetic aperture sonar system. The receiver array is mounted along the ship keel, while the transmitter array is mounted perpendicular to the receiver array. With the synthetic aperture technique, the along-track resolution can be greatly improved. The system often suffers from motion error, which severely degrades the imaging performance. To solve this problem, the imaging algorithm with motion compensation (MC) is proposed. With the presented method, the motion error is first estimated based on overlapped elements between successive pulses. Then, the echo data is processed by using the range migration algorithm based on the phase center approximation (PCA) method, which simultaneously performs the MC with the estimated motion error. In order to validate the proposed sub-bottom profiler and data processing method, some simulations and lake trial results are discussed. The processing results of the real data further indicate that the presented configuration has great potential to find buried objects in seabed sediments.

## 1. Introduction

Nowadays, the exploration of buried sediment in the sea bottom draws researchers’ attention. Using the penetration capability of low frequency signals in water, the sub-bottom profiler [1] is often exploited to detect the structure of soft sediment. Based on matched filtering, the chirp sub-bottom profiler [2,3] uses a chirp signal to obtain high resolution images. The spatial resolution of multibeam sonar [4] is obtained based on the narrow beamwidth. It is often configured with the Mills cross configuration by using a long linear projector aligned with the ship track and a long linear receiver array aligned perpendicular to the receiver array. The transmitter array generates a beam that is narrow in the along-track dimension and wide in the across-track dimension. The receiver array forms multiple beams that are wide in the along-track dimension and narrow in the across-track dimension. By applying different time delays to various receiver signals, the receiver beams can be steered in different directions. The two-way beam patterns are, therefore, narrow in both directions. However, the narrow beamwidth is obtained at the cost of large receiver arrays. The physical length of the receiver array is usually constrained by the sonar platform. Additionally, the resolution decreases with the increase of range. Based on the nonlinear properties of sound propagation, the parametric sub-bottom profiler [5,6] can generate a low frequency via the difference between two transmitted signals. A new signal is used to detect the buried sediment in the sea bottom. However, the efficiency of signal generation from primary to secondary waves is not efficient. In general, sub-bottom profilers do not consider the along-track resolution. For traditional sonar systems, high resolution is obtained at the cost of physically large arrays. Traditional side-scan synthetic aperture sonar (SAS) systems [7,8,9,10] creates high resolution images. With the motion of the sonar platform, a virtual array that is much longer than its physical length can be synthesized by combining multiple pulses with signal processing. Accordingly, high resolution images can be generated. This technique allows us to circumvent the trade-off between the sizes of sonar aperture and along-track resolution. In general, conventional multibeam sonar uses a long receiver array, while the SAS system uses a relatively short receiver array. By using the Mills cross configuration of multibeam sonar and the synthetic aperture technique of the SAS system, this paper introduces a SAS framework called the SAS sub-bottom profiler. This is different from traditional multibeam sonar [6], in that the receiver array is mounted along the keel of a ship, while the transmitter array is mounted perpendicular to the receiver array. Based on this new configuration, the along-track resolution can be obtained for high resolution images. The presented configuration is also different from the side-scan system in [11], as the side-scan configuration is still exploited. Due to insufficient energy, the side-scan SAS system may fail to detect the buried sediment in the sea bottom. With the presented configuration, both the transmitter array and receiver array look downward along the moving path. The presented configuration results in advantageous power budgets, since the seafloor strongly reflects the energy in the incoming direction at the normal incidence rate.

The SAS technique is based on the assumption that the sonar travels in a straight line at a constant speed and depth. In practice, it is impossible to realize this motion. Factors such as waves, tidal currents, and tow boat wake lead to deviation from the nominal trajectory of a SAS system. Generally, motion errors lengthening and reducing the expected time delay seriously affect the SAS imagery. Until now, motion compensation (MC) methods have been commonly grouped into two categories. The first category highly relies on the output of an inertial navigation system (INS) [12,13], providing motion information such as yaw, roll, surge, and sway. However, INS cannot provide micro motion errors, which are close to the sonar wavelength. This issue can be solved by using the other category based on the echo data of a SAS system. Currently, SAS systems consisting of a linear receiver array [14,15,16,17] are often exploited. After transmitting a pulse, the echo data corresponding to multiple receivers is received. Hence, a coarse resolution image is obtained by coherently processing the echo data within each pulse. By cross-correlating two coarse resolution images based on echo data collected from two adjacent pulses [18,19], one can estimate the differential yaw and sway that occurred between the two adjacent pulses. With suitable integration over the full set of recorded echoes, the overall yaw and sway can be estimated. However, it is not efficient to compute coarse resolution images. Based on the overlapped phase centers between adjacent pulses, one can use the correlation between the signal from different receivers coming from the same illuminated scenario. With this method, a displaced phase center antenna (DPCA) [20,21] is used to estimate the sway and yaw based on spatial and temporal coherence properties of the seafloor backscattering. Sonar systems often suffer from six types of motion errors, namely surge, sway, heave, roll, pitch, and yaw errors. Some motion errors have greater effects than others. In this paper, the influence of motion errors on the imagery is first analyzed in detail. Based on the analysis that the heave seriously affects the SAS imaging performance, we estimate the motion error using the overlapped elements between successive pulses. Then, the imaging algorithm with the MC is developed. Herein, the processing of simulated data and real data is used to validate the presented method.

In this paper, a novel sub-bottom profiler based on the Mills cross configuration of multibeam sonar and the synthetic aperture technique of the SAS system is first proposed. Then, the motion error distorting the imaging performance of presented sub-bottom profiler is estimated based on overlapped elements between successive pulses. After that, the range migration algorithm integrated with the MC is presented to improve the imaging performance. This paper is organized as follows. Section 2 introduces the imaging geometry and signal model in the absence of motion error. Section 3 discusses the influence of motion error on SAS imaging results. In Section 4, the motion error is estimated based on overlapped elements between successive pulses, and then the imaging method integrated with the MC is presented. Section 5 processes the simulated data and real data based on the presented method. The last section reports some conclusions.

## 2. Imaging Geometry and Signal Model without Motion Error

**Figure 1:**
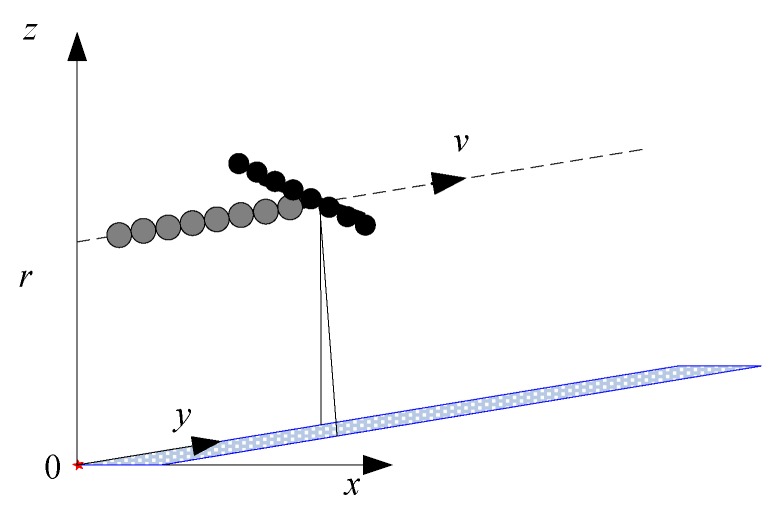
Geometry of the presented sub-bottom profiler.

The new system is based on the combination of a linear array of nadir pointing receivers and conventional SAS technique. The three dimensional (3D) imaging geometry is shown in Figure 1. The receiver array is mounted along the ship moving track. The transmitter array is mounted perpendicular to the receiver array. The system looks downward along the moving path. With this configuration, the transmitter array generates a beam that is narrow in the across-track dimension and wide in the along-track dimension. The seafloor strongly reflects the energy in the incoming direction at a normal incidence rate. In Figure 1, the narrow gridding strip in the horizontal plane denotes the mapping scenario. For clarity, we suppose that there is an ideal target point in the mapping scenario. Its coordinates are (0,0,0). The platform velocity is denoted by *v*, while the sound velocity in water is represented by *c*. The displaced distance between the phase center of the transmitter array and the *i*-th receiver is *d_i_*. Here, i (i∈[1,N]) denotes the receiver index, and *N* denotes the total number of receivers. The first receiver is close to the transmitter, and the last receiver is far away from the transmitter. The height of the sonar system is supposed to be *r*.

At the beginning, the phase center coordinates of the transmitter array are (0, 0, *r*), while the coordinates of the *i*-th receiver are (0, −*d_i_*, *r*). When the moving distance of the transmitter array is *vt*, the coordinates of the *i*-th receiver are (0, *vt* − *d_i_*, *r*). Here, *t* denotes the slow time in the along-track dimension. The echo signal of the *i*-th receiver is the transmitted signal delayed by the two-way time corresponding to the two-way slant range. For the *i*-th receiver, the two-way slant range from the transmitter to the target and then back to the *i*-th receiver is given by

(1) $$R_{i}\left(\tau ,t;r\right)=\sqrt{{\left(v\cdot t\right)}^{2}+r^{2}}+\sqrt{{\left(v\cdot t-d_{i}+v\cdot \tau_{i}\right)}^{2}+r^{2}}$$

where $\tau_{i}$ denotes the accurate propagation time of the transmitted signal [22,23]. Here, $v\cdot \tau_{i}$ represents the moving distance in the along-track dimension during the echo collection; τ is the fast time in the across-track dimension.

With the two-way slant range, the backscattered signal of the *i*-th receiver after demodulation is given by

(2) $$ss_{i}\left(\tau ,t;r\right)=p\left(\tau -\frac{2R_{i}\left(t;r\right)}{c}\right)\exp\left\{-j2\pi \frac{R_{i}\left(t;r\right)}{\lambda }\right\}$$

where p(τ) denotes the transmitted chirp signal and λ represents the wavelength corresponding to the center frequency $f_{c}$. In order to simplify notations, the beam patterns of the transmitter and receiver are not considered, and constants are also discarded.

Inspecting the configuration shown in Figure 1, it can be found that the presented SAS configuration looks downward along the moving path. This is the major difference compared with traditional side-scan SAS systems [11]. Generally speaking, side-scan SAS systems focus on the wide swath, while the SAS sub-bottom profiler only concentrates on sediment penetration with low frequency signals. That is to say, the narrow swath is the main characteristic of the presented configuration. For this reason, the *y*-*z* plane in Figure 1 is considered to be the two-dimensional (2D) imaging geometry for the presented configuration. For simplicity, the medium refraction index is not considered in this paper.

## 3. Influence of Motion Error on Imagery

**Figure 2:**
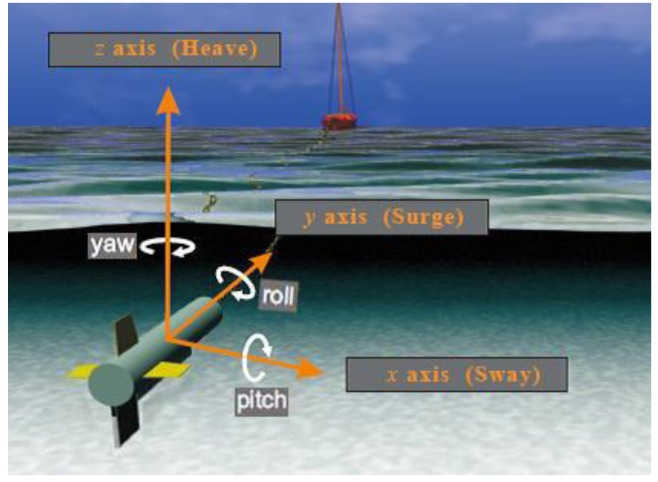
The motion error of the sonar system.

When the sonar platform is towed in water, the system may suffer from the effects of waves, tidal currents, tow boat wake, medium fluctuation, and so on. Due to these reasons, the sonar platform deviates from the nominal path. Under this condition, the SAS system suffers from motion errors, which can severely affect the SAS imagery. In order to easily describe the motion of the sonar platform, a set of three orthogonal axes is placed onto the sonar platform. Figure 2 shows the common terms describing the motion error.

From Figure 2, the sonar system has six possible motion errors [19], which can be further decomposed into translational motion and rotational motion. The translational motion, including the surge, sway, and heave. is defined as variations of a straight, level, and constant moving heights. The surge is a forward or backward shift in the *y* axis. The sway is a side to side translation, and it is a horizontal shift in the *x* axis. The heave is a vertical translation in the *z* axis. The pitch, roll, and yaw belonging to the rotational motion are angular rotations around the *x*, *y*, and *z* axes, respectively. The motion errors can distort the imaging performance. Generally, the motion errors lengthening and reducing the expected time delay would affect the imagery. In this section, simulations are performed to discuss the influence of motion error on the imaging performance. The system parameters are shown in Table 1.

**Figure 3:**
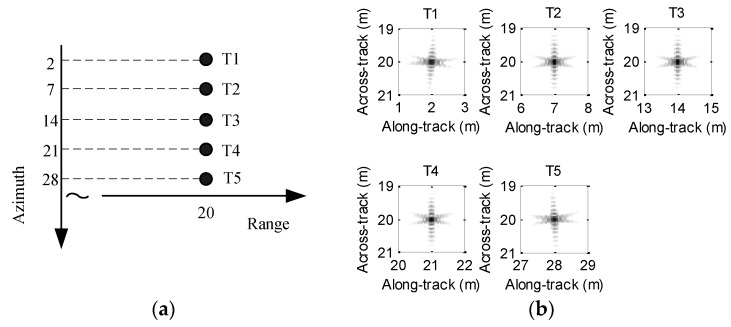
Simulated scenario and imaging result: (**a**) scenario with five-point targets; (**b**) imaging result without motion error.

Generally, the influence of motion error on processing results cannot be thoroughly presented if a single target is chosen. At this point, several targets are usually set in the imaging scenario. For clarity, the imaging scenario is supposed to consist of five ideal targets, which are depicted in Figure 3a. In order to clearly describe the focusing performance, the targets are marked as T1, T2, T3, T4, and T5. Since the time domain algorithms [23,24,25,26] are often viewed as the most precise methods, we can use the imaging results of time domain methods to evaluate other imaging methods. For comparison, the echo data in the absence of motion error is first processed by using the back projection (BP) method [26], and the imaging result is shown in Figure 3b. From Figure 3b, the five targets are well constructed when the echo data is not contaminated by the motion error.

### 3.1. Influence of Sway and Yaw

**Figure 4:**
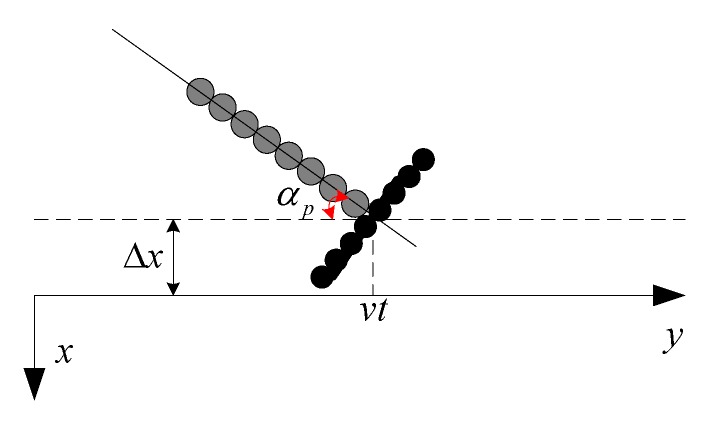
Geometry of sway and yaw in the *x*-*y* plane.

We suppose that the sway deviating from the nominal path is Δx. In Figure 2, the negative yaw is defined as a clockwise rotational movement around the *z*-axis. In Figure 4, the 2D geometry of the sway and yaw in the *x*-*y* plane is depicted. For simplicity, the transmitter phase center is defined as the coordinate origin. Here, $\alpha_{p}$ denotes the yaw and the subscript p is the pulse index.

When the transmitter is located at vt in the *y*-axis, the transmitter coordinates are (Δx,vt,r). When the echo signal is received by the *i*-th receiver, the receiver coordinates are $\left(\Delta x+d_{i}\sin\alpha_{p},vt-d_{i}\cos\alpha_{p}+v\cdot \frac{2r}{c},r\right)$. Here, the moving distance during the signal reception is approximated by $v\tau_{i}\approx v\cdot \frac{2r}{c}$. Therefore, the two-way range from the transmitter to ideal target and then back to the *i*-th receiver is given by

(3) $$\begin{array}{l} R_{i}\left(t;r\right)=\sqrt{\Delta x^{2}+{\left(vt\right)}^{2}+r^{2}}+\sqrt{{\left(\Delta x+d_{i}\sin\alpha_{p}\right)}^{2}+{\left(vt-d_{i}\cos\alpha_{p}+v\cdot \frac{2r}{c}\right)}^{2}+r^{2}}\\ \;\approx 2r+\frac{{\left(vt-\frac{d_{i}}{2}\cos\alpha_{p}+v\frac{r}{c}\right)}^{2}}{r}+\frac{{\left(2v\frac{r}{c}-d_{i}\cos\alpha_{p}\right)}^{2}}{4r}+\frac{\Delta x^{2}+{\left(\Delta x+d_{i}\sin\alpha_{p}\right)}^{2}}{2r}\\ \;\approx 2\sqrt{r^{2}+{\left(vt-\frac{d_{i}}{2}\cos\alpha_{p}+r\frac{v}{c}\right)}^{2}}+\frac{{\left(2v\frac{r}{c}-d_{i}\cos\alpha_{p}\right)}^{2}}{4r}+\frac{\Delta x^{2}+{\left(\Delta x+d_{i}\sin\alpha_{p}\right)}^{2}}{2r} \end{array}$$

Inspecting Equation (3), the error introduced by the sway and yaw is very slight, as the inequality $\frac{\Delta x^{2}+{\left(\Delta x+d_{i}\sin\alpha_{p}\right)}^{2}}{2r}\ll 1$ is always met. We conclude that both motion errors do not degrade the imaging performance. In this section, the influence of sway and yaw on the imaging performance is analyzed individually.

#### 3.1.1. Influence of Sway

We first discuss the influence of sway on the imaging performance under the condition of $\alpha_{p}=0$. Since the deviation is far less than the target range, the inequality |Δx|≪r is always met. Here, |Δx| denotes the sway magnitude. Due to this reason, we conclude that the sway nearly has no effect on the SAS imagery. To validate this conclusion, the simulations are carried out.

**Figure 5:**
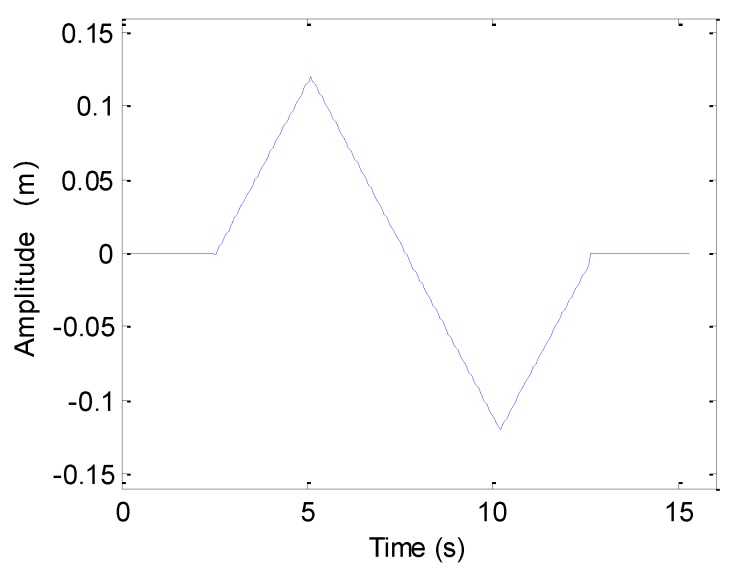
Sawtooth perturbation.

Here, two canonical examples of synthetic aperture motion errors are discussed. We first introduce the sawtooth perturbation into the echo data, and the motion error is shown in Figure 5.

**Figure 6:**
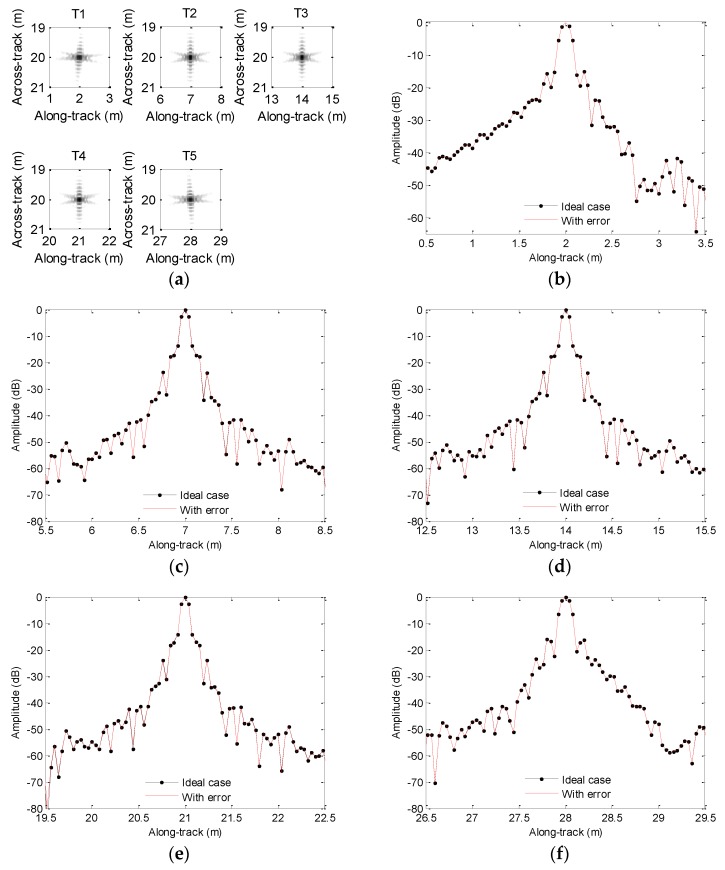
Processing results with the sawtooth sway: (**a**) imaging result; (**b**) slice of T1; (**c**) slice of T2; (**d**) slice of T3; (**e**) slice of T4; (**f**) slice of T5.

Figure 6a shows the imaging results. To visually compare the imaging performance, Figure 6b–f shows the along-track slices of the focused target. In Figure 6, the ideal case denotes the along-track slices without motion error by using the back projection (BP) algorithm [26]. Inspecting Figure 6, the imaging performance with the sawtooth perturbation is nearly identical to that without the motion error. That is to say, the sawtooth perturbation does not affect the imaging performance.

**Figure 7:**
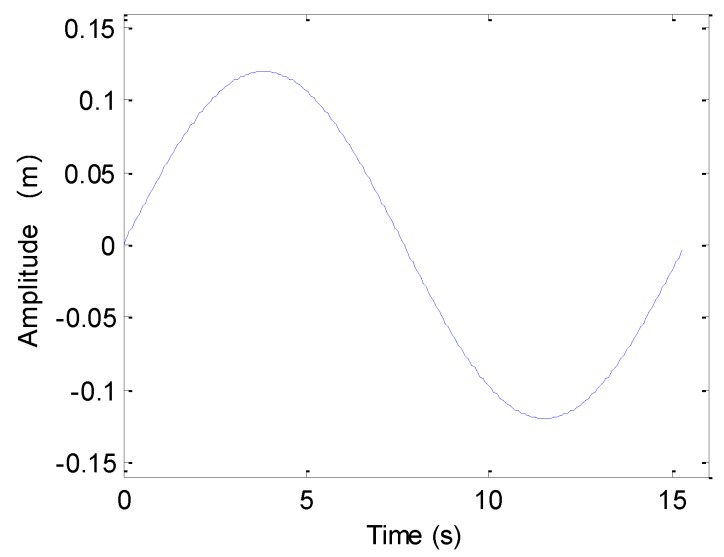
Sinusoidal perturbation.

The next experiment concentrates on the case of sinusoidal perturbation, which is shown in Figure 7.

**Figure 8:**
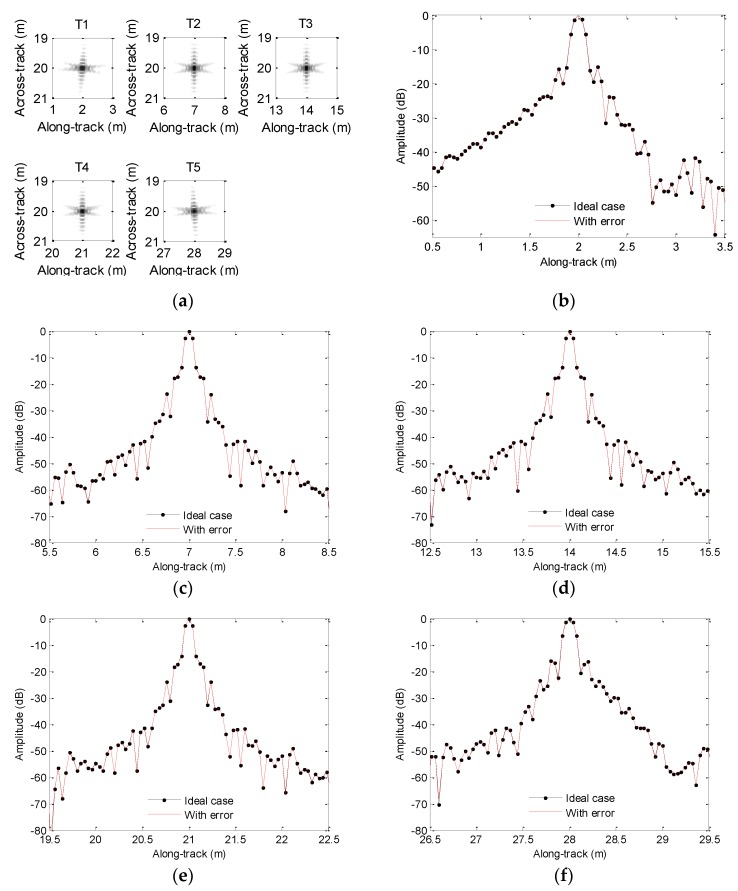
Processing results with the sinusoidal sway: (**a**) imaging result; (**b**) slice of T1; (**c**) slice of T2; (**d**) slice of T3; (**e**) slice of T4; (**f**) slice of T5.

After processing the echo data, the results are shown in Figure 8. From Figure 8, the along-track slices with sinusoidal sway mostly agree with slices in the absence of motion error, and we nearly obtain the same imaging result with the ideal case.

The peak sidelobe level ratio (PSLR), the integral sidelobe level ratio (ISLR) and deviation of along-track coordinates (DAC) are exploited to quantitatively evaluate the imaging performance. Table 2 lists the quality parameters for the two aforementioned experiments.

From Table 2, the PSLR and ISLR with sway error are mostly identical to the corresponding values without the motion error. The along-track coordinates of the ideal target are perfectly reconstructed, even if the echo data is contaminated by the sway. Based on the two aforementioned experiments, we draw a conclusion that the sway does not noticeably affect the imaging performance. Therefore, we can neglect the sway and focus our attention on other motion errors.

#### 3.1.2. Influence of Yaw

**Figure 9:**
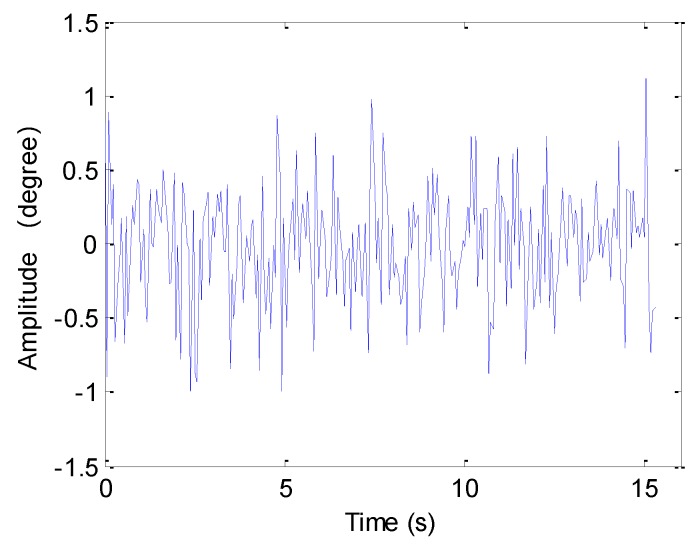
Yaw perturbation.

As Δx=0 was set in Equation (3), the yaw influence on imaging performance is discussed now. It should be noted that the large rotational motion errors are directly obtained from INS. Due to this reason, we mainly concentrate on the microrotation motion error, which is usually beyond INS accuracy. Figure 9 shows the yaw perturbation, which is attributed to the Gaussian processes.

**Figure 10:**
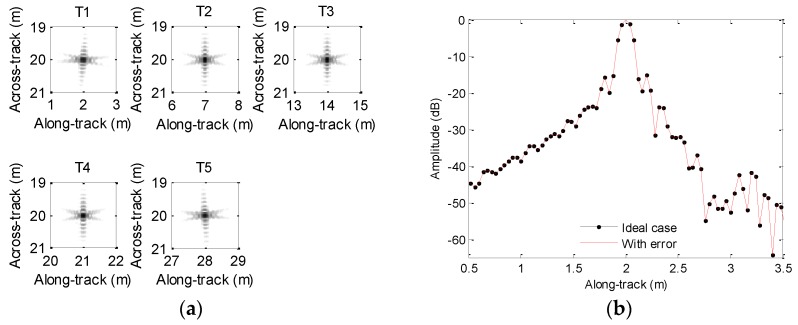

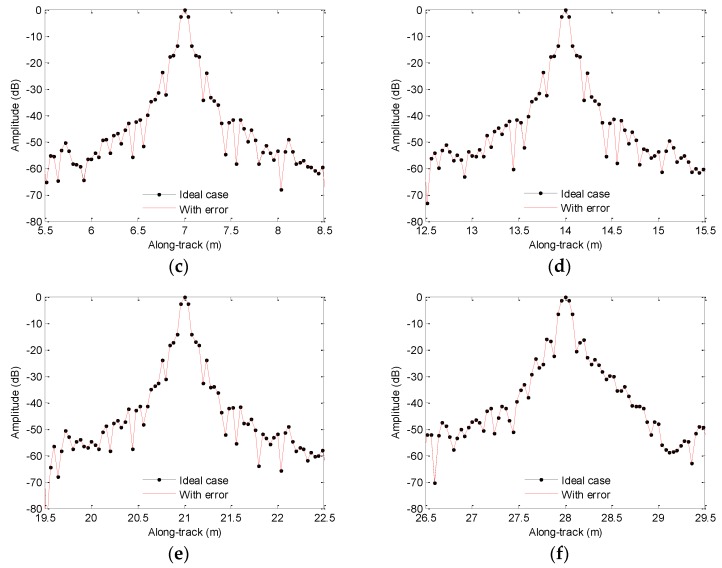
Processing results with the yaw perturbation: (**a**) imaging result; (**b**) slice of T1; (**c**) slice of T2; (**d**) slice of T3; (**e**) slice of T4; (**f**) slice of T5.

The data contaminated by the yaw is directly processed, and the resultant images are shown in Figure 10.

From Figure 10, we find that the yaw perturbation has little influence on the imaging performance. To some degree, it can be found from Equation (3) that the yaw influence is equivalent to that of sway on SAS imaging performance.

Based on simulations, we draw a conclusion that both sway and yaw essentially do not affect the imaging performance. Therefore, both motion errors can be neglected.

### 3.2. Influence of Heave and Pitch

**Figure 11:**
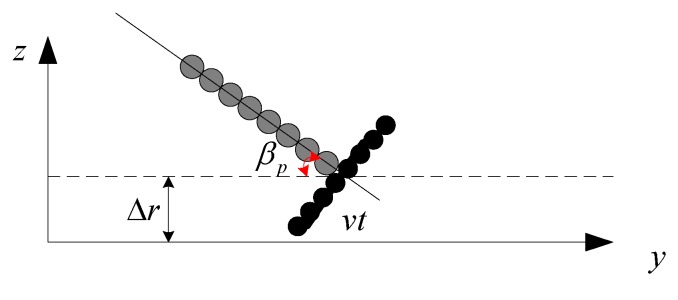
Geometry of heave and pitch in the *y*-*z* plane.

In Figure 2, the positive pitch is defined as a clockwise rotational movement around the *x*-axis. In Figure 11, we depict the 2D geometry of heave and yaw in the *y*-*z* plane. Here, Δr in Figure 11 is supposed to be the heave. For simplicity, the phase center of the transmitter is still defined as the coordinate origin, $\beta_{p}$ denotes the yaw, and subscript p is the pulse index.

When the transmitter is located at vt in the *y*-axis, the transmitter coordinates are (0,vt,r+Δr). The coordinates of the *i*-th receiver are $\left(0,vt-d_{i}\cos\beta_{p}+v\cdot \frac{2r}{c},r+\Delta r+d_{i}\sin\beta_{p}\right)$ when the echo signal is received by this receiver. Therefore, the two-way range from the transmitter to the ideal target and then back to the *i*-th receiver is given by

(4) $$\begin{array}{l} R_{i}\left(t;r\right)=\sqrt{{\left(vt\right)}^{2}+{\left(r+\Delta r\right)}^{2}}+\sqrt{{\left(vt-d_{i}\cos\beta_{p}+v\cdot \frac{2r}{c}\right)}^{2}+{\left(r+\Delta r+d_{i}\sin\beta_{p}\right)}^{2}}\\ \;\approx 2r+\frac{{\left(vt-\frac{d_{i}}{2}\cos\beta_{p}+v\frac{r}{c}\right)}^{2}}{r}+\frac{{\left(2v\frac{r}{c}-d_{i}\cos\beta_{p}\right)}^{2}}{4r}+2\Delta r+d_{i}\sin\beta_{p}\\ \;\approx 2\sqrt{r^{2}+{\left(vt-\frac{d_{i}}{2}\cos\beta_{p}+r\frac{v}{c}\right)}^{2}}+\frac{{\left(2v\frac{r}{c}-d_{i}\cos\beta_{p}\right)}^{2}}{4r}+2\Delta r+d_{i}\sin\beta_{p} \end{array}$$

From Equation (4), it can be seen that the heave and pitch lengthen or reduce the expected time delay. Therefore, the imaging performance must be sensitive to both motion errors. Here, the influence of pith and heave on the imaging performance is also discussed individually.

#### 3.2.1. Influence of Heave

When the condition $\beta_{p}=0$ in Equation (4) is met, we can analyze the heave influence on imaging performance. Inspecting Equation (4), the last term denotes the motion error, which is introduced by heave. With the same magnitude of sway and heave (i.e., |Δr|=|Δx|), the inequality $\frac{\Delta x^{2}}{r}\ll 2\left|\Delta r\right|$ is always met. Slight heave will severely influence the SAS imagery. Using the SAS parameters shown in Table 1 and the BP algorithm [26], we perform simulations to validate this conclusion.

**Figure 12:**
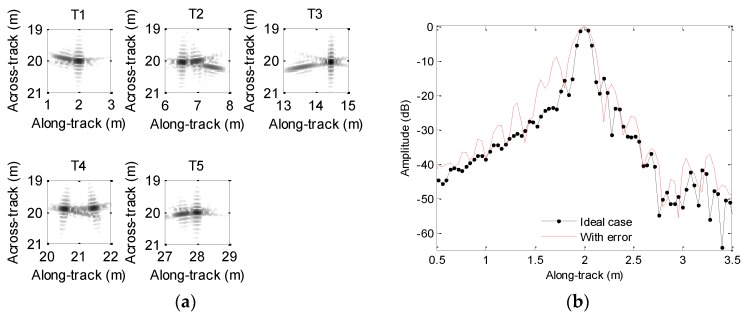

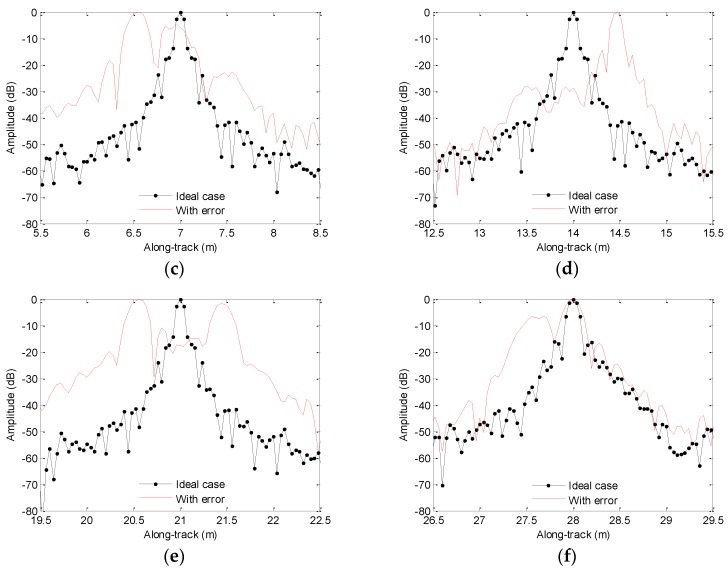
Processing results with the sawtooth heave: (**a**) imaging result; (**b**) slice of T1; (**c**) slice of T2; (**d**) slice of T3; (**e**) slice of T4; (**f**) slice of T5.

We first focus on the processing of echo data when the heave is caused by sawtooth perturbation. The motion error is shown in Figure 5. The processing results and along-track slices are shown in Figure 12. Inspecting Figure 12, all targets are defocused, as the motion error has already lengthened or reduced the expected time delay. The motion error would lead to the deviation of along-track coordinates. Additionally, the sidelobe level is increasing. The focusing results of T1, T3, and T5 enhance this conclusion. Considering T2 and T4, the mainlobe is dramatically influenced by the sidelobe, and imaging results cannot provide useful information about targets. Therefore, the heave severely distorts the imaging performance. That is to say, the heave should be simultaneously compensated when targets are focused.

**Figure 13:**
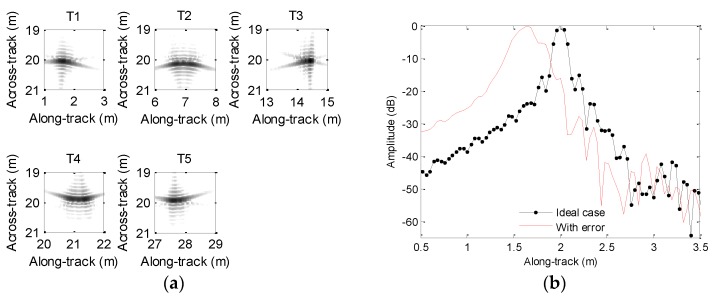

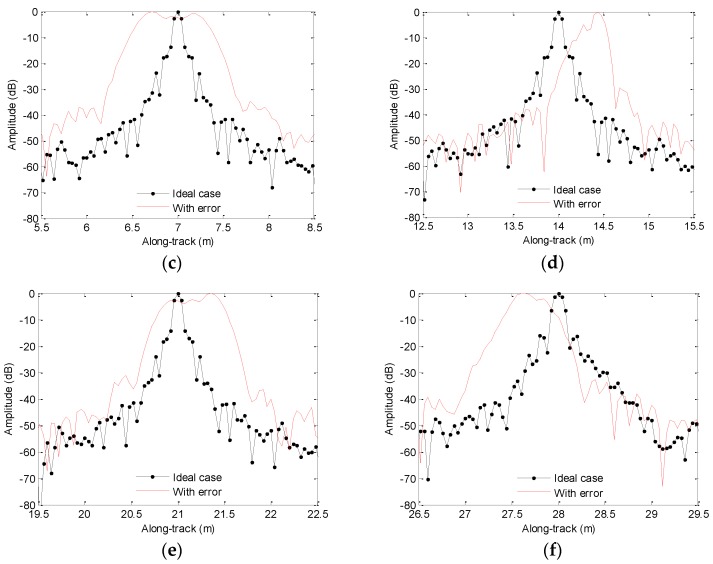
Processing results with sinusoidal heave: (**a**) imaging result; (**b**) slice of T1; (**c**) slice of T2; (**d**) slice of T3; (**e**) slice of T4; (**f**) slice of T5.

Considering the heave caused by sinusoidal perturbation shown in Figure 7, we show the processing results in Figure 13. Inspecting Figure 13, we nearly obtain the same conclusions drawn from Figure 12, because the sinusoidal perturbation also lengthens or reduces the expected time delay.

Table 3 lists the PSLR, ISLR, and DAC when the echo data is contaminated by the heave. Based on Table 3, it can be found that the quality parameters with the motion error are significantly lowered. Besides, the along-track position of the focused target dramatically deviates from that shown in Figure 3. Therefore, the microheave should be estimated and compensated when the SAS imagery is carried out.

#### 3.2.2. Influence of Pitch

**Figure 14:**
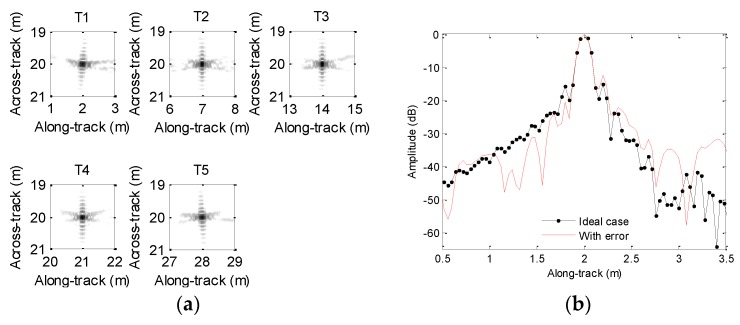

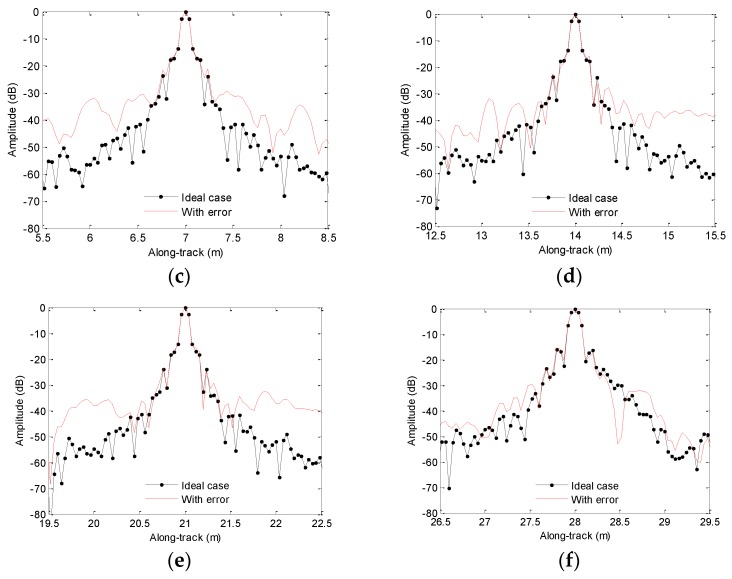
Processing results with pitch: (**a**) imaging result; (**b**) slice of T1; (**c**) slice of T2; (**d**) slice of T3; (**e**) slice of T4; (**f**) slice of T5.

As Δr=0 was set in Equation (3), we now discuss the pitch influence on imaging performance. In Figure 9, the rotational motion error is also considered to be the pitch perturbation. After processing the data contaminated by the pitch error, the resulting images are depicted in Figure 14.

As seen in Figure 14, the pitch does affect the imaging performance. Fortunately, the influence of micropitch is tolerable. Figure 14a further enhances this conclusion. In practice, the large pitch is easily obtained from the INS. However, it is hard to obtain the micropitch, which is beyond the accuracy of INS. Inspecting Equation (4), the influence of pitch on SAS imaging performance is mostly equivalent to that of heave on the SAS focusing performance. At this point, we estimate the average heave for the SAS system in the case of $\beta_{p}=0$. This assumption is reasonable, as the receiver array is very short.

### 3.3. Influence of Surge

**Figure 15:**
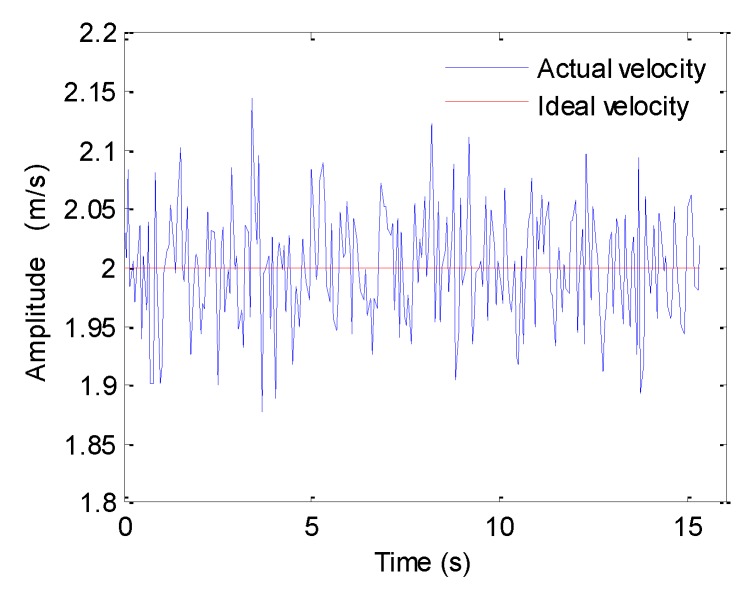
Platform velocity.

The waves, tidal currents, tow boat wake, and medium fluctuation make the platform velocity unstable. For these reasons, the system would suffer from surge, which is a forward or backward shift in the along-track dimension. Under this condition, the sampled data in the along-track dimension is nonuniform [27,28]. Figure 15 shows the platform velocity. Based on sonar parameters shown in Table 1, the ideal velocity satisfying the uniform sampling in the along-track dimension should be 2 m/s. In practice, the platform velocity usually vibrates around the ideal velocity.

**Figure 16:**
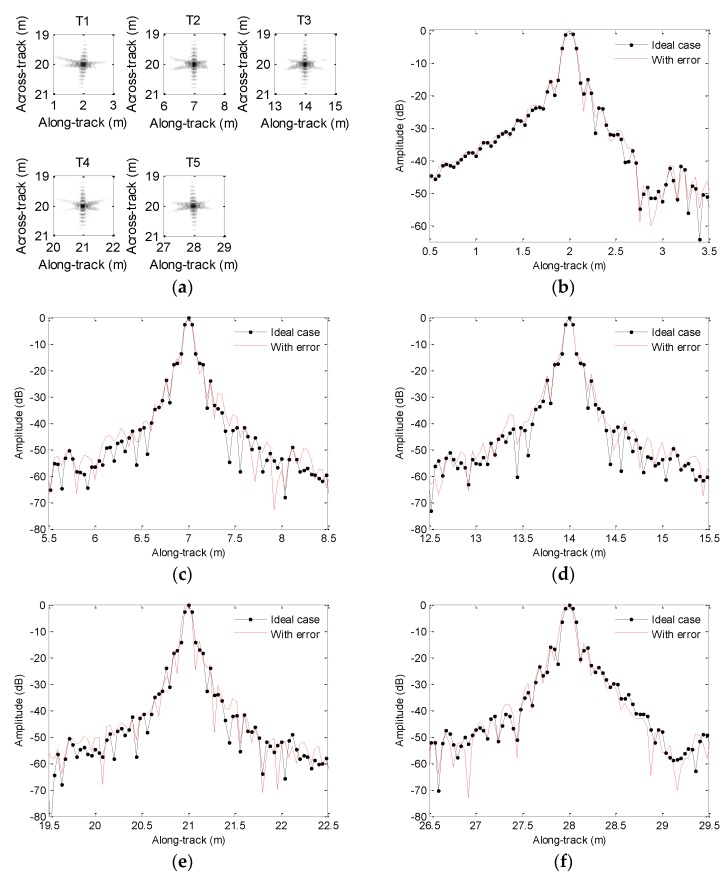
Processing results with surge: (**a**) imaging result; (**b**) slice of T1; (**c**) slice of T2; (**d**) slice of T3; (**e**) slice of T4; (**f**) slice of T5.

After processing the echo data contaminated by surge, the resultant images are shown in Figure 16. It should be noted that the average velocity (i.e., 2 m/s) is used by the imaging algorithm. From Figure 16, the surge has a slight influence on the imaging results. Generally, the imaging algorithm is directly performed when the platform velocity slightly deviates from the ideal one. If the platform velocity largely deviates from the ideal one, the nonuniform sampled data should be coerced into the uniform data before the imaging [27,28].

Inspecting Equations (3) and (4), the yaw and pitch also lead to the surge in the along-track dimension. However, the slight surge does not noticeably affect the imaging performance. At this point, the echo data suffering from the microsurge is still considered to be uniform data. Therefore, we can also neglect the influence of surge on SAS imagery.

## 4. Motion Estimation and Imagery

The sonar system often suffers from motion error, which seriously affects the imaging performance. Therefore, we must estimate and compensate for the motion error. In this paper, the microheave beyond the INS accuracy is directly estimated from the sonar data. With the estimated motion error, we focus the sonar data using the range migration algorithm [29,30] based on the phase center approximation (PCA) method [16], because this algorithm is very efficient compared with the BP algorithm [26].

### 4.1. Estimation of Motion Error

The sonar platform is assumed to travel in a straight line with a constant speed. Due to unknown platform movement, deviations from this straight path often happen in real environments. We first start with the estimation of motion error.

Since the echo signal received by the receiver highly depends on the time delay of the transmitted signal, any change of time delay would degrade the focusing performance. With the SAS configuration shown in Figure 1, heave is the main motion error that directly affects the along-track resolution, which will be discussed in detail. The heave causes the platform to be either lifted up toward the ocean surface or pushed down toward the ocean floor. It has the effect of shortening or lengthening the overall time delay from the moment a pulse is transmitted to the echo from the received target. Since heave equally affects all of the receivers in the case of a multiple receiver system, the extra time delay is mostly identical for each receiver.

**Figure 17:**
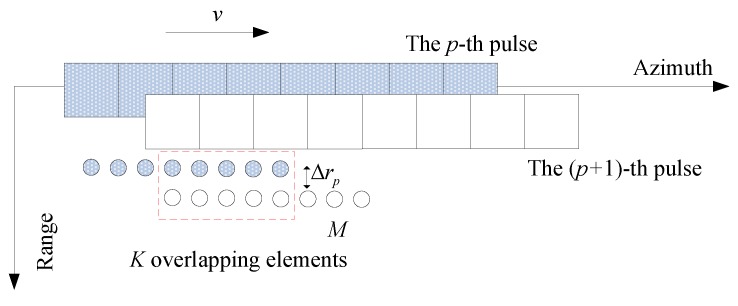
The heave between successive pulses.

From Figure 17, the transmitter and each receiver can be modelled by the transducer located at the midpoint based on the DPCA. K=N−M equivalent elements overlap from pulse to pulse by operating at a speed of $v=\frac{Md}{2PRI}$. Here, PRI represents the pulse repetition interval. The receiver array includes *N* uniformly spaced receivers. The echo of *M* (*M* < *N*) receivers is used to focus the target. In fact, the SAS parameters are chosen in such a way that there is always overlapping of equivalent receiving arrays between two successive pulses in the opposite direction of a subset of equivalent receiving arrays, and there are some equivalent monostatic channels that receive exactly the same signal during two successive acquisitions in the absence of motion errors, regardless of the spatial distribution of elementary scatterers. For simplicity, we assume that the noise has been disregarded and that scatterer geometry and propagation medium have not changed.

In the presence of motion errors, two equivalent receiving arrays between two successive pulses will no longer overlap but form a synthetic interferometric baseline. The change in round-trip travel time to each scatterer will be determined by the projection of the baseline in the radial direction to the scatterer. If the angular spread of this direction within a given sonar resolution cell or a set of resolution cells within a given time window remains small compared to the angular resolution of the interferometric baseline, then the changes in travel time will be the same for all targets in the resolution cell. For this time window, two received signals will, therefore, be nearly identical in shape, but will be received with slightly different delays after transmission. This difference provides an estimate of the line-of-sight motion error.

Considering the influence of heave on the echo signal, the received echo of the *k*-th element is

(5) $$s_{k}(\tau ,p)=\left|s_{k}(\tau ,p)\right|\exp\left\{j\varphi_{p}(\tau ,p)\right\}$$

where k(k∈[1,K]) is the index of overlapped elements. Here, p(p∈[1,P]) is the pulse index, $\left|s_{k}(\tau ,p)\right|=\left|ss(\tau ,Np-N+M+k)\right|$ represents the signal magnitude, and $\varphi_{p}(\tau ,p)$ denotes the signal phase.

We suppose that the sonar receivers suffer from the common motion error $\Delta r_{p}$ for all targets. After transmitting the (*p* + 1)-th pulse, the received signal of the overlapped element is

(6) $$s_{k}(\tau ,p+1)=\left|s_{p}(\tau -\frac{2\Delta r_{p}}{c},p)\right|\exp\left\{j\varphi_{p}(\tau -\frac{2\Delta r_{p}}{c},p)\right\}\exp\left\{-j4\pi f_{c}\frac{\Delta r_{p}}{c}\right\}$$

Since the computer can only obtain the wrapped phase distribution in (−π,π], the measured phase is written as

(7) $$\left[\varphi_{p}(\tau -\frac{2\Delta r_{p}}{c},p)-2\pi f_{c}\frac{2\Delta r_{p}}{c}\right]\mathrm{mod}2\pi$$

where mod(⋅) denotes the modulus operator. After integrating the motion error, the unwrapped phase is obtained.

We now concentrate on the estimation steps of motion error in detail. Inspecting the first term in Equation (4), the *i*-th receiver and transmitter can be approximated by a single transducer located midway between them, as the along-track coordinates of the *i*-th receiver, transmitter, and equivalent transducer are vt, $vt-d_{i}+v\cdot \frac{2r}{c}$, and $vt-\frac{d_{i}}{2}+r\frac{v}{c}$, respectively. This approximation is equivalent to the PCA method [16]. With this approximation, the two-way path from the transmitter to the target and then back to the *i*-th receiver can be approximated by the two-way path between the target and equivalent transducer. However, this introduces range error, which is called the PCA error. The second term in Equation (4) is the PCA error. The last term in Equation (4) is introduced by the heave. Applying a 2D Fourier transformation to Equation (4), we obtain the point target reference spectrum (PTRS) based on the stationary phase of the method [31]. After neglecting the spectrum magnitude, the PTRS is given by

(8) $$SS_{i}\left(f_{\tau },f_{t};r\right)=P(f_{\tau })\exp\left\{j\theta_{i}\right\}$$

with

(9) $$\theta_{i}(f_{\tau },f_{t})=-\frac{4\pi }{\lambda }r\sqrt{{\left(1+\frac{f_{\tau }}{f_{c}}\right)}^{2}-\frac{\lambda^{2}f_{t}{}^{2}}{4v^{2}}}+2\pi f_{t}\frac{r}{c}-\pi f_{t}\frac{d_{i}}{v}-2\pi \left(f_{c}+f_{\tau }\right)\frac{{\left(v\frac{2r}{c}-d_{i}\right)}^{2}}{4cr}-4\pi \left(f_{c}+f_{\tau }\right)\Delta r$$

where $P(f_{\tau })$ is the spectrum of the transmitted signal, $f_{\tau }$ is the instantaneous frequency corresponding to the fast time in the across-track dimension, and $f_{t}$ is the Doppler frequency corresponding to the slow time in the along-track dimension.

Inspecting Equation (9), the first term is similar to the PTRS phase of the monostatic SAS system. The second term is introduced by the stop-and-hop approximation. The target would suffer from the deviation of the along-track coordinates if it were not compensated. The third term denotes the spatial sampling in the along-track dimension. The fourth and last terms are introduced by the PCA error and motion error, respectively.

The heave is estimated by using the data of the overlapped elements between two successive pulses [20,32]. Inspecting Equation (4), the monostatic conversion from multireceiver data to monostatic equivalent data should first be performed. The PCA error that is the second term in Equation (4) should be compensated. Generally, the PCA error is decomposed into the Doppler phase error and microrange migration, which is caused by the spatial displacement of the transmitter and receiver. In order to discriminate this range migration from that introduced by the movement of the sonar system, we name it the bistatic range migration. We start with the compensation of the Doppler phase error introduced by the PCA error. Inspecting Equation (4), the filtering function used for the compensation of Doppler phase error is expressed as

(10) $$H_{1\_i}(\tau ,t;r)=\exp\left\{j\pi f_{c}\frac{1}{2cr}{\left(2\frac{v}{c}r-d_{i}\right)}^{2}\right\}$$

Using the spectrum of the transmitted signal, the pulse compression in the across-track dimension is performed. Examining the second term in Equation (4), the bistatic range migration is a range variant. Considering the wide swath SAS system, the interpolation should be used to deal with the space variance of the bistatic range migration. The presented system is mainly used for sub-bottom detection, and the mapping swath is very narrow. At this point, the compensation of bistatic range migration can also be carried out in the instantaneous frequency domain. The term is given by

(11) $$H_{2\_i}(f_{\tau },t;r)=P^{*}(f_{\tau })\exp\left\{j\pi f_{\tau }\frac{{\left(v\frac{2r_{\mathrm{ref}}}{c}-d_{i}\right)}^{2}}{2cr_{\mathrm{ref}}}\right\}$$

where * denotes the conjugate operation and $r_{\mathrm{ref}}$ represents the reference range, which is often considered to be the height of the sonar platform.

After the monostatic conversion, the echo signal of all overlapped elements within the *p*-th pulse is superposed:

(12) $$s(\tau ,p)=\sum_{k=1}^{K}s_{k}(\tau ,p)$$

The superposition of the echo signal gives us two advantages. One improves the robustness of motion error estimation, and the other improves the signal-to-noise ratio. Based on the same method, we superpose the echo signal of all overlapped elements within the (*p +* 1)-th pulse:

(13) $$s(\tau ,p+1)=\sum_{k=1}^{K}s_{k}(\tau ,p+1)$$

Using s(τ,p) and s(τ,p+1) between two successive pulses, we perform the correlation operation with lag τ=0. This is given by

(14) $$g(0,p)=\sum_{\tau }s(\tau ,p)s^{*}(\tau ,p+1)$$

where Ʃ denotes the summation in the across-track dimension.

Based on Equation (14), the average phase difference for the *p*-th pulse is expressed as

(15) Δϕ⌢(p)=arctan{Im[g(0,p)]Re[g(0,p)]}

where arctan(⋅) denotes the operation of arctangent. Im(⋅) and Re(⋅) are the imaginary and real parts of the complex signal, respectively.

Due to the wrapped characteristic, the phase error shown as Equation (15) is constrained within (−π,π]. The unwrapped phase error is further obtained by integrating the average phase error. This operation is given by

(16) $$\Delta \overset{⌢}{\phi }(p)=\Delta \overset{⌢}{\phi }(p-1)+\Delta \overset{⌢}{\phi }(p),\;\Delta \overset{⌢}{\phi }(p=0)=0$$

With the center frequency $f_{c}$, the time delay error introduced by the platform displacement is expressed as

(17) $$\Delta \tau (p)=\frac{\Delta \overset{⌢}{\phi }(p)}{4\pi f_{c}}$$

With the estimated time delay, the motion error is simultaneously compensated when the SAS imagery is carried out.

### 4.2. Imagery with the MC

Here, the MC is integrated into the SAS imaging algorithm. After the range compression and cancelation of bistatic range migration, we extract the *M* receiver data used for the SAS imagery. Then, we compensate the motion error in the across-track frequency domain. The filtering function is

(18) $$H_{3\_i}=\exp\left\{j4\pi \left(f_{c}+f_{\tau }\right)\Delta r\right\}$$

It should be noted that the variance of time delay in the along-track dimension is ignored due to the narrow swath of the SAS sub-bottom profiler.

After this operation, the M×P receiver data collected with P pulses is arranged in order. The monostatic conversation from the multireceiver data to the monostatic equivalent data is also carried out. With the resultant data, the range migration algorithm [27,28] is used. Applying along-track FT to the data yields the spectrum in the 2D frequency domain. Based on Equation (19), the bulk focusing is performed.

(19) $$H_{4}\left(f_{\tau },f_{t};r_{\mathrm{ref}}\right)=\exp\left\{j\frac{4\pi }{c}r_{\mathrm{ref}}\sqrt{{\left(f_{c}+f_{\tau }\right)}^{2}-f_{t}^{2}\frac{c^{2}}{4v^{2}}}\right\}$$

With this step, the targets in the reference range are correctly focused. However, targets away from the reference range are only partially focused. Therefore, extra steps should be used to handle this issue. The Stolt mapping, which is also called the interpolation in the 2D frequency domain, can deal with this issue. This step remaps the instantaneous frequency axis based on Equation (20).

(20) $$f_{r}=\sqrt{{\left(\frac{2\pi f_{\tau }+2\pi f_{c}}{c/2}\right)}^{2}-{\left(\frac{2\pi f_{t}}{v}\right)}^{2}}-\frac{2\pi f_{c}}{c/2}$$

After this operation, targets away from the reference range are all focused. Then, the inverse Fourier transformation (IFT) in the across-track dimension is performed. After this processing, the along-track offset is corrected by using Equation (21):

(21) $$H_{5}\left(f_{t};r\right)=\exp\left\{-j2\pi f_{t}\frac{r}{c}\right\}$$

The last operation performs the IFT in the along-track dimension. After this step, the high resolution image is obtained.

**Figure 18:**
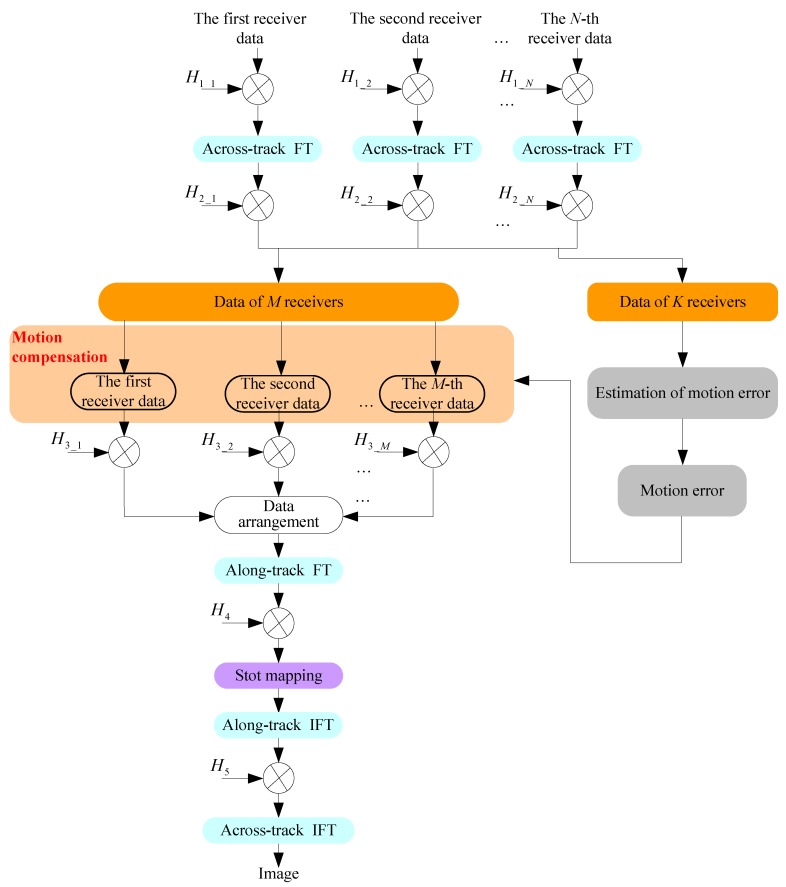
Block diagram of the presented method. FT = Fourier transformation; IFT = inverse Fourier transformation.

Based on processing steps, the block diagram of the presented imaging method is shown in Figure 18.

## 5. Simulation and Real Data Processing

In this section, the simulated data and real data are used to verify the presented sub-bottom profiler and signal processor.

### 5.1. Simulation Results

**Figure 19:**
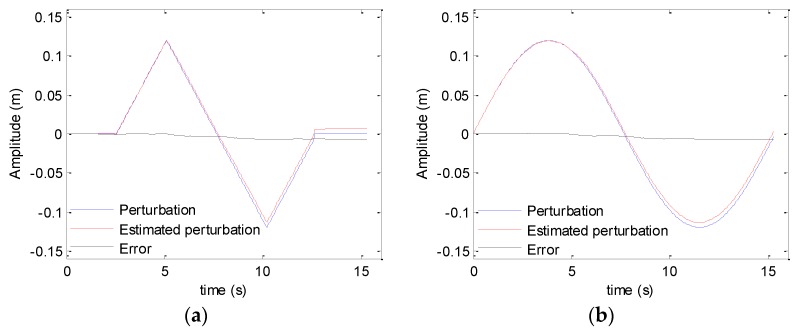
Estimation of motion error: (**a**) sawtooth perturbation; (**b**) sinusoidal perturbation.

This section concentrates on the processing of simulated data. The system parameters can be found in Table 1. The first experiment focuses on the sawtooth heave. With the presented method in Section 4.1, the motion errors shown in Figure 5 and Figure 7 can be estimated. The original perturbation, the estimated error, and the difference between both curves are shown in Figure 19. From Figure 19, it can be seen that the presented method can estimate the motion error.

**Figure 20:**
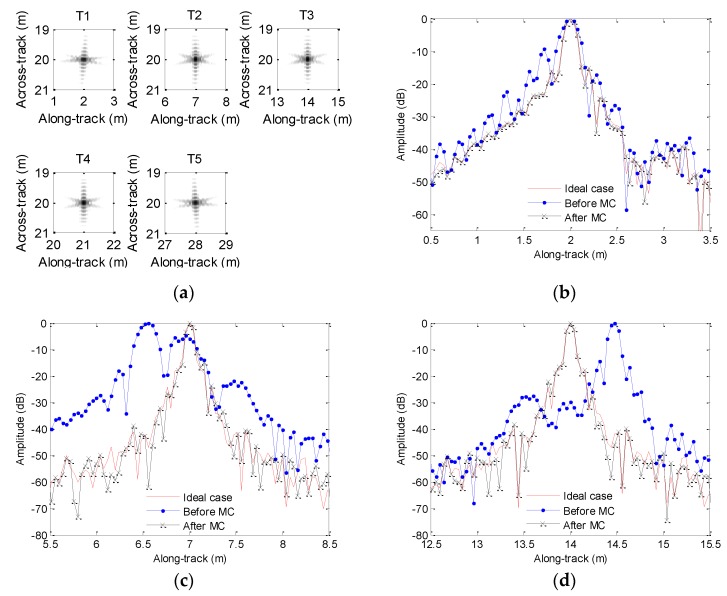

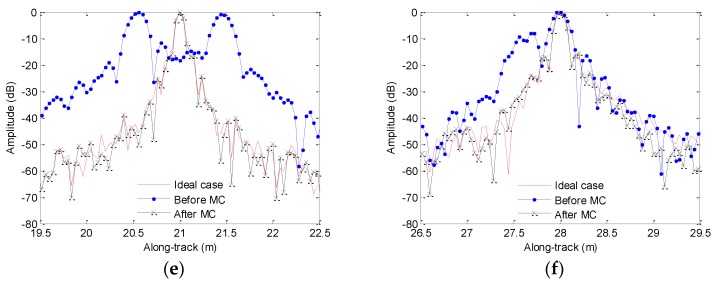
Results for sawtooth heave: (**a**) imaging result; (**b**) slice of T1; (**c**) slice of T2; (**d**) slice of T3; (**e**) slice of T4; (**f**) slice of T5. MC = motion compensation.

In order to compare the imaging performance, Figure 20 depicts the along-track slices of the focusing target. When we process the echo data not contaminated by the motion error based on the method in Section 4.2, we call it the ideal case. From Figure 20, the imaging performance is dramatically degraded if the echo data contaminated by the sawtooth perturbation are directly processed by the imaging algorithm. In Figure 20, slice sidelobes before MC are increasing. The imaging results shown in Figure 20b,c,f enhance this conclusion. Besides, the positions of focused targets deviate from the target positions shown in Figure 3a. The results shown in Figure 20c,d strengthen this conclusion. Furthermore, the ghost targets shown in Figure 20e are introduced by the motion error. Fortunately, the slices after MC mostly agree well with the slices of the ideal case. The presented method nearly gives the same performance as that in the absence of motion error. Therefore, we draw the conclusion that the imaging performance is greatly improved by using the presented method.

Table 4 lists the quality parameters. Based on quality parameters of T4 in Table 4, the PSLR and ISLR before MC differ from the values of the ideal case by about 15 and 24.7 dB, respectively. The DAC is 0.44 m. This conclusion is consistent with the focused results in Figure 20e, as the ghost target is introduced by the motion error. Inspecting quality parameters of other target in Table 4, the PSLR and ISLR before MC are also lowered compared with the corresponding values in the ideal case. The motion error leads to different DACs for various targets. That is to say, the distributed targets may suffer from distortion. Fortunately, the imaging performance is greatly improved by using the presented method. The PSLR and ISLR after MC mostly agree with the corresponding values in the ideal case. With the presented method, all target coordinates are reconstructed.

**Figure 21:**
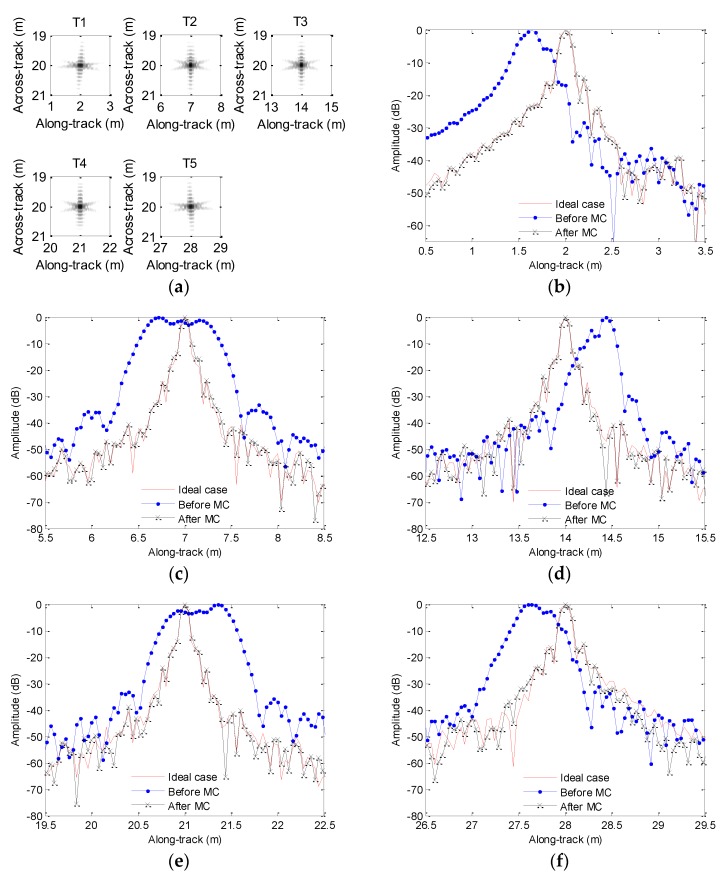
Results for the sinusoidal heave: (**a**) imaging result; (**b**) slice T1; (**c**) slice of T2; (**d**) slice of T3; (**e**) slice of T4; (**f**) slice of T5.

Figure 21 shows the processing results when the echo data is contaminated by the sinusoidal perturbation. In general, the motion error leads to four negative results. The first one is that the reconstructed targets before MC are no longer located at the ideal positions shown in Figure 3a. All focused targets before MC in Figure 21 enhance this conclusion. Since each scatterer owns various DACs, the distributed targets would be distorted. The second result is that the sidelobes are increasing. The slices in Figure 21b,d also strengthens this conclusion. Under this condition, it is very difficult to detect targets with weak reflectivity. The third result is that the ghost targets are introduced. Figure 21c demonstrates this conclusion. The last result is that the mainlobes of slices are broadening. In this circumstance, the resolution is lowered, and it is impossible for the system to distinguish between two neighboring scatterers. With the presented method, the aforementioned four issues can be solved. Inspecting Figure 21a, the presented method can reconstruct the targets, and the focusing performance is nearly identical to that without motion error.

With this experiment, we also present the quality parameters listed in Table 5.

From Table 5, the major difference of PSLR and ISLR for both cases is about 14.4 and 17 dB, respectively. This is consistent with the focused target in Figure 21c. Inspecting the performance of the other target in Table 5, the imaging performance before MC is seriously degraded compared with that of the ideal case. With the presented method, the motion error is well estimated and compensated, and the quality parameters with the presented method are mostly close to the corresponding values in the ideal case.

Based on simulations, we conclude that the presented method can estimate the motion error well. Additionally, the presented imaging method with the MC dramatically improves the focusing performance.

### 5.2. Real Data Processing

**Figure 22:**
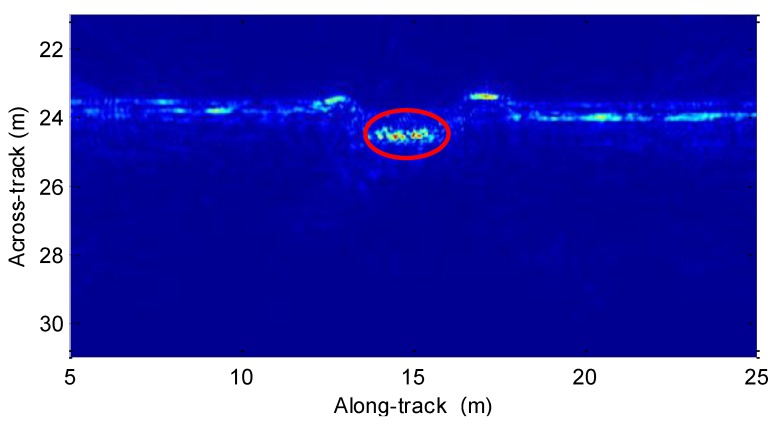
Imaging result without the MC.

In this section, the real data is used to test the presented method. The test was performed in the Zhanghe reservoir. The transmitter array and receiver array have eight elements each. The diameter of each element is 0.08 m. The chirp signal is used by the system. The center frequency is 12 kHz, while the signal bandwidth is 7 kHz. The platform velocity is about 2 m/s. The pulse repetition interval is 0.06 s. Without the MC, the echo data is directly processed by the imaging processor presented in Section 4.2. The resulting image is shown in Figure 22. From Figure 22, we find that the targets circled by the red line are defocused.

**Figure 23:**
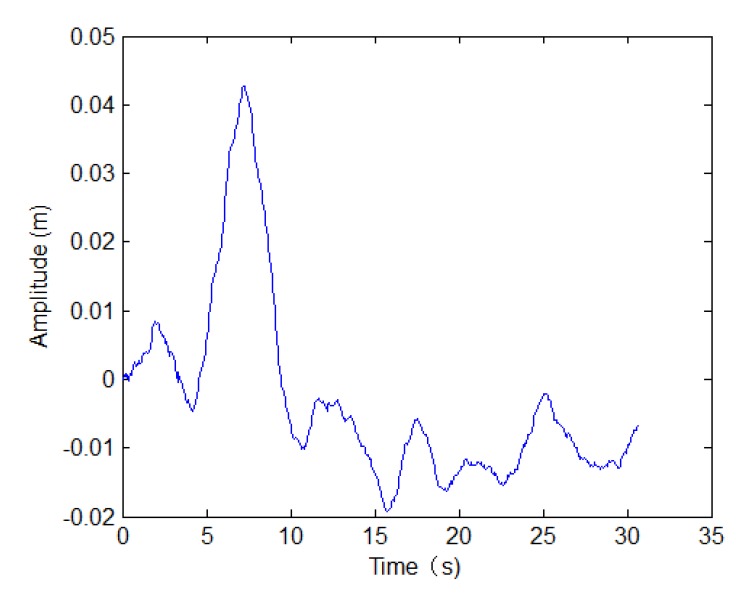
Motion error with real data.

Inspecting Figure 22, the targets circled by the red circle are distorted by the uncompensated motion error. Based on the method in Section 4.1, we can estimate the motion error, which is shown in Figure 23.

**Figure 24:**
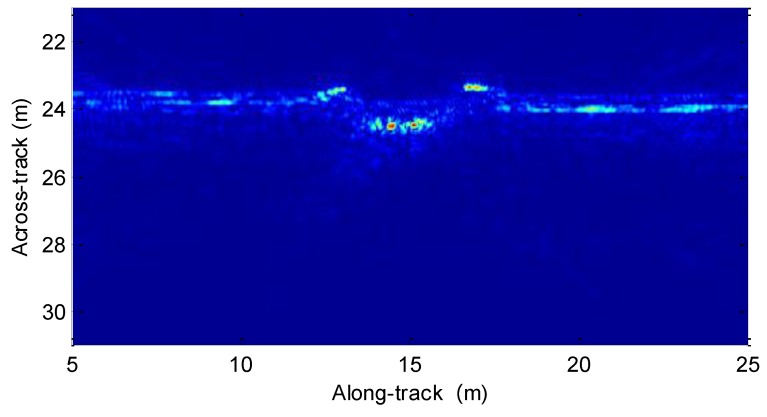
Imaging result with the presented method.

With the estimated motion error and imaging method in Section 4.2, the real data is processed. The resultant image is shown in Figure 24. Inspecting Figure 24, the defocused targets in Figure 22 are well reconstructed by using the presented method. The imaging performance is greatly improved by using the presented method in this paper. Additionally, the sediment penetration with the low-frequency signal is successful. That is to say, the targets in the sediment can be detected by exploiting the presented SAS sub-bottom profiler.

## 6. Conclusions

This paper first proposes a novel sub-bottom profiler based on the Mills cross configuration of multibeam sonar and the synthetic aperture technique of the SAS system, and then presents a signal processor improving the imaging performance of the presented sub-bottom profiler. On the one hand, the presented sub-bottom profiler exploits the Mills cross configuration of multibeam sonar. The receiver array is mounted along the ship’s moving track, while the transmitter array is mounted perpendicular to the receiver array. This is the major difference between the presented system and multibeam sonar. On the other hand, the synthetic aperture technique of the SAS system is used by the presented system. The sub-bottom profiler presented in this paper looks downward along the moving path, while the traditional SAS systems use the side-scan configuration. Compared with traditional side-scan SAS systems, the transmitter array generates a beam that is narrow in the across-track dimension and wide in the along-track dimension. With the downward-looking configuration, the seafloor strongly reflects the energy in the incoming direction at the normal incidence rate. Furthermore, the synthetic aperture technique allows us to circumvent the trade-off between the sonar aperture size and along-track resolution, as a virtual array that is much longer than its physical length can be synthesized based on the motion of the sonar platform and coherently signal processing. Compared with traditional sub-bottom profilers, the presented system can generate high resolution images in the along-track dimension based on the synthetic aperture technique. Additionally, the signal energy can be efficiently used for sediment penetration with the presented system. At this point, the presented system is very suitable for sub-bottom detection.

The presented sub-bottom system often suffers from motion errors, which play an important role in the imaging performance. In general, motion errors lengthening and reducing the expected time delay would affect the imagery. This paper discusses the influence of motion error on the imaging performance, and indicates that except for heave, the motion errors have nearly no effect on the processing results. Considering the serious influence of heave, an error estimation method based on overlapped elements between successive pulses is presented. Then, a range migration algorithm integrated with MC is discussed. To validate the presented sub-bottom profiler and signal processor, the simulated data and real data based on the presented sub-bottom profiler are processed. The processing results of the simulated data show that the heave can be efficiently estimated by using the presented motion estimation method. Besides, the processing results are greatly improved based on the presented signal processor. The processing results of the real data show that the presented system does penetrate the sub-bottom. The results further indicate that the presented sub-bottom profiler has great potential in sub-bottom detection. Moreover, the results after MC are improved compared with the results before MC. This further validates the signal processor discussed in this paper. 

Our next task is to consider the medium refraction index when it comes to the imagery. In addition, other robust motion error estimation methods should be taken into account to improve the performance of the signal processor.

## Figures and Tables

**Table 1:** The Parameters of the new sub-bottom profiler.

Parameters	Value	Units
Center frequency	15	kHz
Bandwidth	7.0	kHz
Platform velocity	2.0	m/s
Receiver length in along-track dimension	0.08	m
Length of receiver array	0.48	m
Transmitter length in along-track dimension	0.16	m
Pulse repetition interval	0.06	s

**Table 2:** Quality parameters with the sway. Note: PSLR = peak sidelobe level ratio; ISLR = integral sidelobe level ratio; DAC = deviation of along-track coordinates.

	Ideal Case		Sawtooth Error			Sinusoidal Error		
	PSLR/dB	ISLR/dB	PSLR/dB	ISLR/dB	DAC/m	PSLR/dB	ISLR/dB	DAC/m
T1	−15.10	−5.15	−15.08	−5.15	0	−15.03	−5.15	0
T2	−15.58	−11.00	−15.39	−10.98	0	−15.54	−10.97	0
T3	−15.58	−11.02	−15.44	−11.01	0	−15.52	−11.02	0
T4	−15.59	−11.19	−15.47	−11.17	0	−15.48	−11.18	0
T5	−15.18	−6.27	−15.14	−6.27	0	−15.08	−6.26	0

**Table 3:** Quality parameters with heave.

	Ideal Case		Sawtooth Error			Sinusoidal Error		
	PSLR/dB	ISLR/dB	PSLR/dB	ISLR/dB	DAC/m	PSLR/dB	ISLR/dB	DAC/m
T1	−15.10	−5.15	−8.85	−0.99	0	−5.26	3.86	0.36
T2	−15.58	−11.00	−4.60	4.08	0.04	−0.88	7.95	0.28
T3	−15.58	−11.02	−14.18	−7.32	0.52	−4.83	0.99	0.56
T4	−15.59	−11.19	−1.55	2.93	0.44	−2.32	4.34	0.36
T5	−15.18	−6.27	−6.36	0.83	0	−2.08	5.25	0.40

**Table 4:** Quality parameters with the sawtooth perturbation.

	Ideal Case		Before MC			After MC		
	PSLR/dB	ISLR/dB	PSLR/dB	ISLR/dB	DAC/m	PSLR/dB	ISLR/dB	DAC/m
T1	−15.45	−5.23	−9.31	−3.93	0	−15.12	−5.17	0
T2	−15.83	−11.20	−4.63	1.35	0.44	−13.89	−10.45	0
T3	−15.89	−11.18	−17.29	−7.38	0.52	−15.46	−11.06	0
T4	−15.91	−11.39	−0.98	13.32	0.44	−14.74	−10.94	0
T5	−15.30	−6.33	−7.61	−0.52	0	−14.71	−6.28	0

**Table 5:** Quality parameters with the sinusoidal perturbation.

	Ideal Case		Before MC			After MC		
	PSLR/dB	ISLR/dB	PSLR/dB	ISLR/dB	DAC/m	PSLR/dB	ISLR/dB	DAC/m
T1	−15.45	−5.23	−5.62	1.12	0.36	−14.67	−4.94	0
T2	−15.83	−11.20	−1.10	5.76	0.28	−14.15	−11.19	0
T3	−15.89	−11.18	−4.92	−2.32	0.56	−15.30	−11.07	0
T4	−15.91	−11.39	−2.43	1.86	0.64	−15.50	−10.69	0
T5	−15.30	−6.33	−2.57	2.61	0.40	−14.60	−5.99	0
